# Cognitive Impairment in Multiple Sclerosis With Regards to Disease Duration and Clinical Phenotypes

**DOI:** 10.3389/fneur.2019.00261

**Published:** 2019-03-20

**Authors:** Bruno Brochet, Aurélie Ruet

**Affiliations:** ^1^Service de Neurologie, CHU de Bordeaux, Bordeaux, France; ^2^Team Glia-Neuron Interactions, Neurocentre Magendie, INSERM U1215, Université de Bordeaux, Bordeaux, France

**Keywords:** multiple sclerosis, neuropsychology, cognition, phenotypes, cognition

## Abstract

The relationships between cognitive impairment that exist during the clinical course of multiple sclerosis (MS) remain poorly described. The effect of disease duration has been studied in a few longitudinal cohorts and some cross-sectional studies that suggest that cognitive deficits tend to extend with disease duration. However, the effect of disease duration seems to be confounded by the effect of age. At the pre-clinical stage, cognitive deficits have been observed in patients with radiologically isolated syndromes, and their profile is similar than in clinically isolated syndromes (CIS) and relapsing-remitting MS (RRMS). The frequency of cognitive impairment tends to be higher in RRMS than in CIS. In these phenotypes, slowness of information processing speed (IPS) and episodic verbal and visuo-spatial memory deficits are frequently observed, but executive functions, and in particular verbal fluency, could also be impaired. More frequent and severe deficits are reported in SPMS than in RRMS with more severe deficits for memory tests, working memory and IPS. Similarly to what is observed in SPMS, patients with primary progressive MS (PPMS) present with a wide range of cognitive deficits in IPS, attention, working memory, executive functions, and verbal episodic memory with more tests and domains impaired than RRMS patients. Altogether these data suggested that not only the duration of the disease and age play an important role in the cognitive profile of patients, but also the phenotype itself, probably because of its specific pathological mechanism.

## Introduction

The relationships between cognitive impairment (CI) associated with multiple sclerosis (MS) that exist during the clinical course of the disease remain poorly described. When considering the prevalence of CI in the different phenotypes, the respective effects of disease duration and age (and consequently the accumulation of pathology) and of the clinical phenotypes (meaning the different pathological mechanisms underlying these phenotypes) have to be considered. These two dimensions overlapped largely, since in relapsing-onset MS the clinical phenotypes such as clinically isolated syndromes (CIS), relapsing-remitting (RR), and secondary progressive (SP) occur successively.

Methodological issues have to be taken into account when comparing the different studies. First, the NP tests could vary notably between studies. The number of tests, the domains studied, and the psychometric properties of the tests used could affect the results. Second, the definition of CI could also vary; for example, the number of NP scores need to be abnormal and different statistical thresholds were used. In this paper we provide details about the main studies, summarized in tables.

## Cognitive Impairment and Disease Duration

The impact of disease duration on CI has been a matter of debate for many years. This question has been addressed in a few longitudinal studies (1–4), but also in several cross-sectional studies taking disease duration as a covariate (5–8). Table 1 summarizes the longitudinal studies. In a long-term controlled study, Amato et al. (3) and (4) examined 50 MS patients with short disease duration and 70 matched healthy controls (HC). After 10 years, impairment was confirmed for short-term verbal memory, abstract reasoning and linguistic abilities, but attention and short-term spatial memory were also involved (4). This study suggests that as the disease progresses, cognitive deficits tend to extend. Moreover, the proportion of patients who were cognitively preserved decreased over time from 74% at baseline to 44% after 10 years, while the proportion of patients with mild or moderate impairment tended to increase. Early cross-sectional studies concluded with a weak correlation between CI and disease duration (5, 6), or no correlation (7). In a large cross-sectional study including 1,500 MS patients evaluated by computerized NP testing, Achiron et al. (8) studied the effect of disease duration and observed that the proportion of CI increased over 25 years. In another study performed in 168 patients examining the different phenotypes using the Brief-Repeatable Battery of NP tests (BRB-N), an effect of disease duration was observed on all tests (9). A recent multi-center study in a large sample of 1,040 patients with MS tested using the BRB-N and the Stroop test, showed an association of CI with disease duration but also age and disability (10). However, when adjusting disease duration and clinical course to age and disability, the association with CI was no longer significant but it is quite obvious that age and disease duration are strongly associated.

**Table 1:** Controlled longitudinal studies on cognitive impairment in MS.

**References**	**N MS patients**	**N HS**	**Phenotype/DD**	**Duration**	**NP tests**	**Outcome**
Jennekens-Schinkel et al. (1)	33	18	13RR/20 progressive DD: 16 ± 9.9 y	4 y	Specific battery	Variable deterioration in a few patients.
Kujala et al. (2)	42 (20 CP, 22 CI at baseline)	35	9RR/3SP/11PP DD:8.7 ± 6 y	2.8 y [2–3.9]	Mild deterioration battery	2/20 CP and 17/22 CI deteriorated
Amato et al. (3)	49 (50 at baseline) 37/50 CP 4 mild CI 9 moderate CI	70	38RR, 6SP (44 RR at baseline)/5PP (6 at baseline) DD: 1.6 ± 1.6 y at baseline	4.53 ± 1.15 y	Specific battery	25/49 CP 16 mild CI 8 moderate CI
Amato et al. (4)	45 (50 at baseline)	65 (70 at baseline)	26RR, 14SP; 5PP	10 y	Specific battery	20/45 CP 15 mildly CI 10 moderately CI

*N, number; CP, cognitively preserved; CI, cognitively impaired; RR, relapsing-remitting, SP, secondary progressive; PP, primary progressive; DD, disease duration; y, years*.

## Cognitive Impairment According to Clinical Phenotype

It is difficult to compare studies performed in different clinical phenotypes in different settings, with various NP batteries. Studies evaluating MS patients with different phenotypes using a similar methodology are necessary for comparing CI according to these phenotypes. However, the demographic characteristics of the different phenotypes, such as age and gender in particular, are different, and this needs to be taken into account by using appropriate controls.

### Radiologically Isolated Syndromes (RIS)

At the pre-clinical stage, in subjects in whom lesions typical for MS were discovered on magnetic resonance imaging (MRI) performed in another purpose, the so-called RIS, CI has been observed with a similar cognitive profile than in RRMS affecting information processing speed (IPS) and memory (11, 12). So far, only small studies are available, and it is not possible to conclude on the prevalence of CI in RIS.

### Clinically Isolated Syndromes

Two of the earliest studies conducted on CI in the MS spectrum were, in fact, in patients with optic neuritis, one of the most common type of CIS (13) and in CIS in general (14, 15). Many studies have been performed since, but only controlled studies with a healthy control group assessed with the same battery (or recent normative data of the same battery in the same country) are valid for evaluating the prevalence. Table 2 summarizes results of the main studies. The prevalence varies from one study to another, according to selection characteristics (all CIS or CIS with dissemination in space and/or in time, disease duration, lesion load which reflects the duration of the pre-clinical stage, etc.). The study with the highest frequency (57%) (16) was performed in a population of selected CIS patients with dissemination in space on MRI, according to McDonald's et al. criteria (21). The number of NP scores studied was higher than in other studies, fulfilling the criterion of two impaired tests more easily. The study with the smaller prevalence (12.3%) (23) was the only one using the minimal assessment of cognitive function in multiple sclerosis (MACFIMS). Other studies using the BRB-N (Table 2) found frequencies between 18 and 34%, the two studies with the highest figures including patients with longer disease duration.

**Table 2:** Frequency of cognitive impairment (CI) in patients with CIS compared with matched HC or national normative data.

**References**	**CIS *n***	**HS *n***	**% CI CIS**	**Definition CI**	**NP battery**	**DD**
Feuillet et al. (16)	40^***^	30	57	≥2 tests ≤ 2SD	BRB-N + other tests	2.89/0.5 (1–3) months
Potagas et al. (17)	33	43	27.3	>2 scores < 1.64 SD (5th percentile)	BRB-N	1.0 (1.5) years
Zipoli et al. (18)	61	Normative data	25	≥2 tests ≤ 2SD	BRB-N + Stroop	< 3 months
Khalil et al. (19)	44	Normative data	18.2	>1 test (z score < 1.64) (5^th^ percentile)	BRB-N	0.2 (0.1–0.8) years
Reuter et al. (20)	97^*^,^**^	55	20	≥2 scores < 1.64 SD (5th percentile)	BRB-N	5.0 (1–16, 21) months
Vitterbo et al. (22)	100	Normative data	21	≥2 scores < 1.5 SD	BRB-N	< 12 months
Uher et al. (23)	81^**^	134	12.3	≥2 scores < 1.5 SD	MACFIMS	< 4 months
Ruano et al. (10)	167	Normative data	34.5%	≥2 scores < 1.64 SD in ≥2 domains	BRB-N	1.4 (2.2) years

*CIS, clinically isolated syndromes; CI, cognitive impairment; NP, neuropsychological; DD, disease duration; BRB-N, Brief-Repeatable Battery of neuropsychological tests; MACFIMS, Minimal Assessment of Cognitive Function in Multiple Sclerosis. SD, standard deviation*.

* *Oligoclonal bands in cerebrospinal fluid*.

** *>1 lesion brain or cord magnetic resonance imaging*.

*** *Dissemination in space on magnetic resonance imaging*.

The profile of CI in CIS is characterized by slowness of IPS and episodic verbal and visuo-spatial memory deficits (10, 20, 22, 24). In patients with very short disease duration, isolated impairment of IPS was reported (19). In a recent study, a sample of 41 CIS patients, compared to a matched sample of healthy controls (HC), very limited CI was observed, significant only for IPS and visuo-spatial memory (25). In this group of patients, the lesion load was very small, suggesting that they were at a very early stage of the disease.

Executive functions (EF) and, in particular, verbal fluency could also be impaired (10, 20, 22). However, inhibition and switching seems to be preserved in CIS (24). Working memory (WM) impairment has been shown by event-related potential study (26) or eye-tracking oculomotor testing (27). Table 3 presents the frequency of impairment of the different domains in the largest studies using BRB-N.

**Table 3:** Frequency of impairment of different cognitive domains in CIS (BRB-N).

**References**	**N CIS**	**IPS**	**Memory**	**EF**	**WM**
Reuter et al. (20)	97	SDMT 20%	SRT LTS 15% SRT DR 28% SPART 20% SPART DR 17%	WLG (P) 28% WLG (S) 20%	PASAT 3 22%
Vitterbo et al. (22)	100	SDMT 7%	SRT LTS 7% SRT DR 9% SPART 7% SPART DR 2%	WLG 22%	PASAT 3 7%
Ruano et al. (10)	167	IPS^*^ (SDMT/PASAT) 41%	Verbal learning^*^ (SRT) 27% Visuo-spatial^*^ (SPART) 14.5%	EF^*^ (WLG, Stroop) 42%	

*CIS, clinically isolated syndromes; IPS, information processing speed; EF, executive functions; WM, working memory. SDMT, Symbol-Digit modalities test; SRT, Selective Reminding test; LTS, Long-term storage; DR, delayed recall; SPART, Spatial Recall test; PASAT, Paced-Auditory Serial Addition test; WLG, Word List Generation test (P, phonemic; S, semantic)*.

* *At least one test score impaired*.

### Relapsing-Remitting MS

The frequency of CI in RRMS has been measured in many studies, mainly in samples from neurological departments or specialized MS clinics with various mean disease duration, but these studies were rarely controlled. Few studies focused on the early stages of RRMS and on community-based samples. In a controlled study of consecutively enrolled and newly diagnosed RRMS patients [mean disease duration 24.33 (26.49) months (SD)] referred from community-based neurology practices, the frequency of CI was 45% (≥2 scores <1.64 SD of HC scores) (28). A large multi-center study included 550 RRMS untreated with disease-modifying therapies (DMT), with an EDSS ≤4 and mean disease duration of 5.0 (5.3) years (SD), found CI in 34.9% of patients (≥2 scores <1.64 SD) (29). In another large multi-center study in specialized centers using a large battery [461 RRMS patients excluding patients referred for cognitive testing, mean disease duration of 75 months (24–210)], the prevalence of CI was 31% (≥2 scores <1.64 SD) (30). A population-based study in Sicily, showed CI in 36.9% of RRMS patients, with a mean disease duration of 8.0 ± 3.3 years, using the BRB-N and the Stroop test (at least three positive tests involving at least two different domains) (31).

Several studies compared the prevalence of CI in CIS and RRMS and are summarized in Table 4. Although the frequency tends to be higher in RRMS, the differences were not significant. One limitation of these studies is that age at onset was higher in the CIS samples than in the RR samples, suggesting possible selection bias (10, 19).

**Table 4:** Frequency of cognitive impairment (CI) in patients with CIS compared with patients with RRMS.

**References**	**CIS *n***	**RR *n***	% CI^*^ CIS	%CI^*^ RR	**Age CIS**	**Age RRMS**	**DD CIS (years)**	**DD RRMS (years)**
Potagas et al. (17)	33	75	27.3	40.0	34.7 (8.7)	34.3 (8.9)	1.0 (1.5)	6.2 (4.9)
Khalil et al. (19)	44	80	18.2	21.3	33.9 (10.0)	37.0 (9.6)	0.2 (0.1–0.8)	8.1 (4.2–13.8)
Ruano et al. (10)	167	759	34.5	44.5	33.9 (9.8)	39.9 (10.2)	1.4 (2.2)	11.2 (8.4)

*CIS, clinically isolated syndromes; RR, relapsing-remitting; DD, Disease duration; CI, cognitive impairment*.

* *Definition of CI in each study (see Table 1)*.

The cognitive profile in RRMS is very similar to the one observed in CIS, with deficits mainly in IPS, verbal and visuo-spatial memory impairment, and EF (studied by verbal fluency tests in the above studies). However, one study found a lower global cognitive index *z* score (meaning more impaired) in RR vs. CIS patients (19). Another study found a higher effect in RR than in CIS for verbal memory assessed by the selective Reminding Test (SRT) (17). Curiously, the largest study showed in logistic regression model an association between EF impairment and phenotype (CIS vs. RR), illustrating a more frequent verbal fluency impairment in CIS than RRMS (10).

### Secondary Progressive MS

The epidemiology of secondary progressive MS (SPMS) has changed dramatically in the past 15 years, probably in relation to the availability of DMT. Epidemiological studies in patients studied in the 80s' reported that up to 75% of RRMS patients convert to SPMS after 30 years (32), although recent studies showed that the delay of conversion to SPMS was prolonged since the availability of DMT (33, 34), and, therefore, the proportion of converting patients, decrease. However, CI seems to be frequent in SPMS. Few studies estimate the frequency of CI in SPMS and compared the CI in SPMS with other phenotypes. In a study of 45 consecutively recruited SPMS patients, CI (≥2 tests <1.5SD of HC) was diagnosed in 55.6% (35), Another study using computerized testing found 80% of patients with CI out of 30 with SPMS (36). This figure was close to the results of another study reporting 82.8% of CI (>33% of measures <5th percentile of HC), in a sample of 29 patients with SPMS tested by the BRB-N, as compared to 40.0% in a sample of 75 RRMS patients (17). The recent multi-center Italian study reported a high prevalence of CI in 79.4% out of 74 SPMS patients (10).

A study compared the cognitive performances in 28 patients with SPMS to 28 patients with primary progressive MS (PPMS) and 20 HC (37). The CI was not substantially different between the two phenotypes. In a larger study, 71 SPMS patients were compared with RRMS, PPMS and HC, and the results found more severe deficits in SPMS with high contrast estimates between SPMS and RRMS for memory tests, working memory (paced-auditory serial addition test, PASAT), and IPS (symbol-digit modalities test, SDMT) (38). Severity of CI was rather similar between SPMS and PPMS. In a study in 101 patients with various phenotypes, it was found that patients with SPMS were at least two-fold more frequently impaired than patients with RRMS and long disease duration in IPS, EF, verbal fluency, verbal episodic memory, working memory and visuo-spatial construction (39).

In the study cited above by Dackovic et al. (9), patients with PPMS or SPMS were more frequently impaired than those with CIS and RRMS in all cognitive tests in the BRB-N (9).

### Primary Progressive MS

CI has long been considered rare in PPMS (40), which was considered to mainly affect the spinal cord. However, more recent studies have shown that this not the case. The MAGNIMS study analyzed a sample of 191 PPMS or transitional progressive MS patients recruited in specialized centers, 63 out of them being paired to HC. In this study, 28.6% of patients were diagnosed as cognitively impaired with rigorous criteria (≥3 tests <2SD) (41). Few studies have compared, with a similar methodology, selected samples of patients with RRMS and PPMS. They found more frequent impairment in PPMS than in RRMS patients. In one study, CI was diagnosed in 56.5% out of 23 PPMS patients (17). The larger Dutch study found that CI was more severe in PPMS than in RRMS but similar between SPMS and PPMS (38). The recent multi-center Italian study reported 91.3% of CI in 40 PPMS patients (10). However, these studies did not use separate control groups, and the greater mean age of patients with progressive MS could explain the differences with RRMS. In a recent study comparing PPMS and RRMS patients using a unique methodology and an adequate HC sample, PPMS patients presented a wide range of cognitive deficits in IPS, attention, working memory, EF, and verbal episodic memory, whereas the impairment in RRMS patients was limited to IPS, attention, and working memory in comparison with their respective matched HC (42). Besides the fact that CI in PPMS patients concerned more NP scores and more domains than in RRMS patients, PPMS patients had more severe cognitive deficits than RRMS patients. The differences on NP performance between PPMS and RRMS patients were observed after taking into account age and sex by using *z* scores based on the data from matched HC but also after controlling for EDSS. IPS was the most frequently impaired cognitive domain in both PPMS and RRMS patients, but the two cognitive domains, which differed between these two types of MS, were verbal episodic memory and EF with respect to the frequency. In that study, patients with PPMS performed more poorly than HC on 16 of 23 NP scores (69.6%), whereas patients with RRMS exhibited lower NP performance on 5 of 23 scores (21.7%), compared with their matched HC.

## CI in Different Phenotypes and Pathophysiology

In the long-term, CI evolution, considered as a whole, could be related to the progression of both gray matter (GM) and WM pathology. A longitudinal study conducted over 17 and a half years showed that CI progressed continuously paralleled by atrophy and lesion accumulation (43). These results are in agreement with another study of 202 long-standing MS patients in which CI was correlated with brain atrophy and diffuse white matter damage, irrespective of the phenotype (44). However, the role of GM pathology seems to be preponderant as suggested by a 13-year longitudinal study of 73 MS patients, showing that baseline disease duration and average GM magnetization transfer ratio (MTR) were the two only independent variables associated with cognitive deterioration (45).

For a better understanding of the role of the different mechanisms involved in CI, it could be worth studying specific cognitive domains separately and focusing on early stages when all mechanisms could be more easily disentangled. Indeed, when considering the two more frequent cognitive processes impaired at the early stage of the disease, IPS, and memory, different mechanisms could be discussed. It has been suggested that deficits in these two cognitive functions could progress differently in early RRMS patients, IPS being more impaired initially but progressing at a slower rate than memory (46). The different kinetics of CI evolution for these two functions may be explained at least in part by different mechanisms. At this early stage of the disease, the role of focal lesions, network disruption, or GM vulnerability has been suggested. IPS depends on the integrity of large-scale cortical integrative processes, which involve long-distance white matter projections which can be impaired due to diffuse demyelinating injury in patients (focal lesions) and the axonal pathology related to these lesions (28, 46). In early RRMS, the correlation of CI with lesion load is no longer significant when diffused white matter pathology [normal appearing white matter magnetization transfer ratio (28) or Diffusion tensor imaging (DTI) (47) metrics] is taken into account, suggesting that disconnection plays an important role in CI, mainly in IPS deficits. The involvement of several key brain regions has been shown, contributing to these deficits, such as the thalamus (48, 49), the cerebellum (50, 51), and default mode network (52).

Episodic memory impairment is associated with deep GM injury in the limbic system, in particular the hippocampi and the basal ganglia (53, 54). The growing role, along the disease course, of GM pathology spreading initially in regions with more vulnerability in deep GM and the ^cortex^ (55) and leading to progressive brain atrophy (43), could explain the deterioration of memory or other domains like EF.

However, atrophy is the late consequence of either Wallerian degeneration secondary to axonal transections in lesions or direct inflammation in the GM. DTI shows microstructural abnormalities in the GM that precedes atrophy. Experimental and human studies in CIS using DTI have shown that a selective vulnerability of some GM regions, for instance, hippocampus and some cortical areas, could occur (56). A role for inflammatory injury of the GM, in the hippocampi, associated with microglial infiltration leading to synaptic defects, has been demonstrated in an animal model of early MS (56). This early GM involvement seems to selectively affect some GM areas with more vulnerability, like the dentate gyrus of the hippocampi (57). Meningeal inflammation has been shown to be associated with inflammatory damage in the GM and seems to be more extended as long as the disease progress (58). This predominant GM pathology in progressive stages could explain the cognitive profile observed in these stages. For instance, EF impairment, which is more prominent in progressive stages, has been shown to be related with frontal cortical pathology (59). The importance of synaptic dysfunction in the development of CI has been recently underlined (60).

## Conclusion

Although some similarities could be observed in the cognitive profile of the different phenotypes in MS, with predominant involvement of IPS and episodic memory, some differences could be observed. Memory and EF deficits seem to be more frequent as long as the disease progresses, although IPS seems to appear early and progress slowly. The highest prevalence of CI and its profile in the progressive forms of the disease as compared to the relapsing forms is in accordance with the recent pathological findings underlining the major involvement of the gray matter in these phenotypes (61).

## Author Contributions

All authors listed have made a substantial, direct and intellectual contribution to the work, and approved it for publication.

### Conflict of Interest Statement

BB reports personal fees and non-financial support from biogen-idec, grants from merck-serono, personal fees and non-financial support from novartis, personal fees and non-financial support from genzyme, grants, personal fees and non-financial support from teva, grants, and non-financial support from bayer, outside the submitted work. AR reports grants from TEVA, during the conduct of the study; personal fees and non-financial support from Novartis, personal fees and non-financial support from Biogen, grants, personal fees and non-financial support from TEVA, grants and non-financial support from Roche, grants and non-financial support from Merck, grants and non-financial support from Genzyme, non-financial support from Medday, grants from Bayer, outside the submitted work.

## Abbreviations

MS: multiple sclerosis
CIS: clinically isolated syndrome
RRMS: relapsing-remitting MS
SPMS: secondary progressive MS
CI: cognitive impairment
HC: healthy controls
NP: neuropsychological
BRB-N: Brief-Repeatable Battery of NP tests
RIS: radiologically isolated syndrome
IPS: information processing speed
MACFIMS: Minimal assessment of cognitive function in multiple sclerosis
EF: executive functions
WM: working memory
DMT: disease-modifying therapies
PPMS: primary progressive MS
PASAT: paced-auditory serial addition test.
